# Risk factors for drug-resistant epilepsy according to sex: a cohort study conducted in a middle-income country

**DOI:** 10.1055/s-0046-1827051

**Published:** 2026-08-13

**Authors:** Ignacio Lagger, Sol Pacha, Eliana Garino, Oscar Martinez, Eduardo Knorre, Glenda Ernst

**Affiliations:** 1 Pontificia Universidad Católica Argentina, Consejo Nacional de Investigaciones Científicas y Técnicas, Instituto de Investigaciones Biomédicas, Buenos Aires, Argentina.; 2 Hospital General de Agudos Dr. Teodoro Álvarez, Departamento de Neurología, Buenos Aires, Argentina.; 3 Hospital Británico de Buenos Aires, Departamento de Neurología, Buenos Aires, Argentina.; 4 Hospital Británico de Buenos Aires, Comité Científico, Buenos Aires, Argentina.

**Keywords:** Drug Resistant Epilepsy, Sex, Risk Factors, Epilepsy, Seizures

## Abstract

**Background:**

Drug-resistant epilepsy (DRE) affects 30% of patients with epilepsy. Although multiple risk factors (RFs) are recognized, there are few studies that explore the differences in clinical presentation, etiology and RFs considering the sex, especially in Latin America.

**Objective:**

To analyze sex differences in clinical features, outcomes and RFs for DRE in patients enrolled in 2 hospitals of Buenos Aires between 2020 and 2022.

**Methods:**

Multicenter retrospective cohort study. Clinical variables, epilepsy characteristics, and complementary studies of patients over 18 years with epilepsy were collected. Descriptive analyses, bivariate comparisons (Chi-squared, *t* -test/Mann-Whitney U test) and logistic regressions were applied to adjust for confounding factors.

**Results:**

A total of 122 patients were included, of whom 44 had DRE. The prevalence of DRE was slightly higher in men than in women (39.6 vs. 33.3%). Male sex showed 4 RFs for DRE: personal history (odds ratio [OR]: 7.82; 95%CI: 1.54–39.51), magnetic resonance imaging (MRI) with abnormal findings (OR: 4.40; 95%CI: 1.26–15.41), symptomatic etiology (OR: 4.77; 95%CI: 1.44–15.77), and time of evolution (OR: 1.05; 95%CI: 1.00–1.06). In women, time of evolution was the only RF for DRE (OR: 1.04; 95%CI: 1.00–1.08).

**Conclusion:**

There are sex differences in clinical, etiological, and imaging features of DRE. It is important to incorporate a sex perspective into the initial assessment, as this could contribute to the early identification of patients with higher risk of DRE. Particularly in men with clinical factors associated with higher risk, it would be advisable to implement earlier and more intensive follow-up strategies.

## INTRODUCTION

Epilepsy is a common neurological disease affecting 1% of the world's population, being characterized by a predisposition to recurrent seizures. 1 The estimated mean incidence varies between countries, being 43.4 per 100 thousand people for industrialized countries, and 68.7 per 100 thousand in developing countries. 2 The estimated prevalence is of 2.7 to 7.1 per 1 thousand people in developed countries and 2.2 to 22.2 per 1 thousand in low- and middle-income countries. 3

Although multiple antiseizure drugs (ASDs) are available, approximately 30% of people with epilepsy continue to have seizures despite receiving the correct medication, defining these patients as drug-resistant. 4 The International League Against Epilepsy (ILAE) defines epilepsy as drug-resistant (DRE) when the person with epilepsy persists with seizures despite receiving at least two correctly selected ASDs at maximum doses (in monotherapy or in combination), which were not discontinued due to adverse effects. 5

Multiple factors have been described that compromise response to medication, one of them being gender, due to the action of hormones on brain tissue. 6 7 8 The relationship between these factors and the incidence of DRE is still controversial.

Some authors suggest that the incidence of DRE is higher in women than in men because steroid hormones and corticosteroids may act on inflammation, brain development, and transporter expression. 8 9 10 Both inflammation and brain development would increase excitatory activity by generating new epileptogenic connections and ongoing inflammation. On the other hand, by increasing the metabolism and excretion of ASDs at the hepatic and renal level, ASD levels in brain tissue would decrease, thereby increasing the risk of DRE. 11

However, there is controversy in these assertions, as other studies find that the incidence of DRE is higher in men, because symptomatic etiologies (head injury, stroke) tend to be more frequent in men and are more associated with drug resistance than cryptogenic and undetermined causes. 12

This disparity in findings suggests that the relationship between sex and drug resistance in epilepsy may be influenced by multiple factors, including differences in brain biology, response to ASDs and distribution of underlying etiologies.

Epilepsy has a major negative impact not only biologically, but also on quality of life, 13 with the latter being greater in patients with DRE. 14 The aim of the study was to determine the risk factors (RFs) associated with DRE according to sex, analyzing their impact on the clinical course of patients. Understanding these factors could help to optimize individualized therapeutic management and improve the quality of life of people with epilepsy.

## METHODS

### Study design

A multicenter, retrospective, cohort study was conducted in Buenos Aires, Argentina, between March 2020 and April 2022. The study was conducted in the Hospital General de Agudos Dr. Teodoro Álvarez (protocol number: 744) and Hospital Británico (protocol number: 2468), after receiving approval from the ethics committees of both centers. All patients voluntarily signed an informed consent form.

### Patient selection

Patients aged 18 years and older, diagnosed with epilepsy according to the ILAE, and followed for at least 2 years were included. Those diagnosed with nonepileptic psychogenic seizures, psychiatric illness, and/or with follow-up time under 2 years were excluded.

### Variables

Demographic variables, family and personal history (antecedent of traumatic brain injury, central nervous system [CNS] infection, febrile seizure, drugs, family history), age at epilepsy diagnosis, time of evolution (time between diagnosis and inclusion in the study, expressed in years) seizure and epilepsy type, and medication used were collected. Complementary studies were also considered to determine the etiology and type of epilepsy (neuroimaging, electroencephalogram). Variables were grouped according to sex, following the Sex and Gender Equity in Research (SAGER) 15 guidelines.

### Statistical analysis

Demographic and clinical variables were first summarized using descriptive statistics. Continuous variables were reported as medians with interquartile ranges (IQRs), and categorical variables as mean and percentages. The normality of continuous variables was examined using the Shapiro–Wilk test. To compare characteristics between drug-resistant epilepsy (DRE) and nondrug-resistant epilepsy (n-DRE), the Mann–Whitney U test was used for continuous variables, and Fisher's exact test for categorical variables, given the sample size distribution.

A univariate analysis was conducted to explore potential associations between each clinical factor and the presence of DRE. Variables significantly associated with DRE in the univariate analysis ( *p* < 0.05) were selected for inclusion in the multivariate model. Before constructing the multivariate model, the pattern of missing data across variables was evaluated to ensure randomness; cases with missing data were not imputed.

A multivariate logistic regression model was then performed to identify independent risk factors for DRE, stratified by sex. Model selection was optimized using a stepwise (bidirectional) regression procedure, aiming to maximize model fit while minimizing overfitting. At each step, variables were removed based on statistical significance, and the Bayesian information criterion (BIC) was applied to limit the number of predictors retained in the final model. Model diagnostics included assessment of multicollinearity (variance inflation factors), evaluation of influential observations and outliers, and analysis of residuals.

The normality of residuals was verified using the Shapiro–Wilk test and visual inspection through QQ plots. Model stability was assessed using linear least squares estimation. The final logistic regression models reported odds ratios (ORs) with 95%CIs. The two-sided significance level (α) was set at 0.05. To control for multiple testing, the final models incorporated false discovery rate (FDR) adjustment, using the Benjamini–Hochberg procedure, expressed as false discovery rate (FDR) LogWorth values. Furthermore, the Prism (GraphPad Software), v. 8.0.1, and Medcalc (MedCalc Software Ltd), v. 12.2.1, were used in the analysis ( **Supplementary Material** – available at https://www.arquivosdeneuropsiquiatria.org/wp-content/uploads/2026/04/ANP-2025.0327-Supplementary-Material.docx ).

## RESULTS

### Population characteristics

A total of 122 patients were included, among whom 44 had a diagnosis of DRE (36.06%) and 78 of n-DRE (63.93%). Demographic characteristics were assessed in the general population ( Table 1 ) as well as according to sex ( Tables 2 3 ). Although the prevalence of drug resistance was higher in men than in women, the difference was not statistically significant (47.72 vs 41.02%: *p* = 0.56).

**Table 1:** Characteristics of patients

Patients' characteristics (n = 122)	Drug-resistant epilepsy	Nondrug-resistant epilepsy	*p-* value
**Total (n)**	44	78	
**Age (years): median (interquartile range 25–75)**	43 (34.25–53.5)	41 (31.75–53)	0.99
**Male sex: n (%)**	21 (47.72)	32 (41.02)	0.56
**Personal history: n (%)**	**42**	**74**	
Risk factor			0.55
1 or more	27	43	
0	15	31	
Traumatic brain injury	6 (13.63)	11 (14.28)	0.9
Meningitis	0 (0)	4 (5.19)	0.295
Febrile seizure	9 (20.45)	2 (2.59)	**0.0018**
Drugs	1 (2.27)	0 (0)	0.36
Family history	7 (15.90)	19 (24.67)	0.35
Others	13 (29.54)	21 (27.27)	0.83
**Time of evolution (years): median (interquartile range 25–75)**	22 (14.5–39)	16 (7–24)	**0.0041**
**Epilepsy type: n (%)**	**44**	**78**	
Generalized	16 (36.36)	42 (53.84)	0.08
Focal	28 (63.63)	34 (43.58)	**0.039**
Unknown	0 (0)	2 (2.56)	0.53
**Location of epileptogenic zone: n (%)**	**44**	**78**	
Frontal	4 (9.09)	7 (8.97)	0.9
Temporal	17 (38.63)	17 (21.79)	0.059
Parietal	1 (2.27)	1 (1.28)	0.9
Occipital	0 (0)	2 (2.56)	0.53
Insular	1 (2.27)	1 (1.28)	0.9
Mesial	9 (20.45)	0 (0)	**0.0001**
Undetermined	21 (47.72)	50 (64.10)	0.08
**Seizure type: n (%)**	**44**	**78**	
Generalized	17 (38.63)	47 (60.25)	0.0591
Focal impaired awareness	24 (54.54)	27 (34.61)	**0.0371**
Focal aware	3 (6.81)	4 (5.12)	0.7
Unknown	0 (0)	0 (0)	0.9
**Neuroimaging: n (%)**			
Magnetic resonance imaging (n = 107)	**42**	**65**	
Abnormal	26 (61.90)	23 (35.38)	**0.0098**
Normal	16 (38.09)	42 (64.61)	
Computed tomography (n = 38)	**11**	**27**
Abnormal	4 (36.36)	8 (29.62)	0.71
Normal	7 (63.63)	19 (70.37)	
Electroencephalogram: n (%)	**40**	**70**	
Abnormal	31 (77.5)	47 (67.15)	0.28
Normal	9 (22.5)	23 (32.85)	
**Etiology, n (%)**	**44**	**78**	
Symptomatic	25 (56.81)	28 (35.89)	0.036
Idiopathic/Undetermined	19 (43.18)	50 (64.10)	

Note: Other risk factors include CNS infections, pregnancy complications, and birth complications. The *p* -values in bold indicate statistical significance ( *p* < 0.05).

**Table 2:** Characteristics of the male patients

Patients' characteristics (men = 53)	Drug-resistant epilepsy	Nondrug-resistant epilepsy	*p* -value
**Total: n (%)**	21 (39.62)	32 (60.37)	
**Age (years): median (interquartile range 25–75)**	43 (32–57)	40.5 (29.25–51.25)	0.96
**Personal history: n (%)**	22	28	
Risk factor			**0.016**
1 or more	19 (83.36)	15 (53.57)	
0	3 (13.63)	13 (46.42)	
Traumatic brain injury	5 (22.72)	6 (21.42)	0.73
Meningitis	0 (0)	2 (7.14)	0.5
Febrile seizure	4 (18.18)	2 (7.14)	0.2
Drugs	1 (4.54)	0 (0)	0.4
Family history	4 (18.18)	8 (28.57)	0.74
Others	9 (40.90)	8 (28.57)	0.23
**Time of evolution (years): median (interquartile range 25–75)**	22 (13.25–31.5)	15 (3–24)	**0.04**
**Epilepsy type: n (%)**	**21**	**32**	
Generalized	7 (33.33)	17 (53.12)	0.17
Focal	14 (66.66)	14 (43.75)	0.15
Unknown	0 (0)	1 (3.12)	0.9
**Location of epileptogenic zone:n (%)**	**21**	**32**	
Frontal	2 (9.52)	3 (9.37)	0.9
Temporal	8 (38.09)	8 (25)	0.36
Parietal	1 (4.76)	0 (0)	0.39
Occipital	0 (0)	0 (0)	0.9
Insular	0 (0)	0 (0)	0.9
Mesial	3 (14.28)	0 (0)	0.056
Undetermined	10 (47.61)	21 (65.62)	0.25
**Seizure type: n (%)**	**21**	**32**	
Generalized	8 (38.09)	19 (59.37)	0.16
Focal impaired awareness	12 (57.14)	12 (37.5)	0.16
Focal aware	1 (4.76)	1 (3.12)	0.9
**Neuroimaging: n (%)**			
Magnetic resonance imaging (n = 46)	**20**	**26**	
Abnormal	14 (70)	9 (34.61)	0.18
Normal	6 (30)	17 (65.38)	
Computed tomography (n = 16)	**6**	**10**	
Abnormal	4 (66.66)	2 (20)	0.11
Normal	2 (33.33)	8 (80)	
Electroencephalogram: n (%)	**18**	**30**	
Abnormal	13 (72.22)	21 (70)	0.9
Normal	5 (27.77)	9 (30)	
**Etiology: n (%)**	**21**	**32**	
Symptomatic	15 (71.42)	11 (34.37)	**0.011**
Idiopathic/Undetermined	6 (28.57)	21 (65.62)	

Notes: Other risk factors include central nervous system infection, pregnancy complication, and birth complication. The *p* -values in bold indicate statistical significance ( *p* < 0.05).

**Table 3:** Characteristics of the female patients

Patients' characteristics (women = 69)	Drug-resistant epilepsy	Nondrug-resistant epilepsy	*p* -value
Number of patients (%)	23 (33.33)	46 (66.66)	
**Age (years): median (interquartile range 25–75)**	43 (32–57)	43 (35.75–53)	0.9
**Personal history: n (%)**	20	46	
Risk factor			0.17
1 or more	8 (40)	28 (60.86)	
0	12 (60)	18 (39.13)
Traumatic brain injury	1 (5)	5 (10.86)	0.65
Meningitis	0 (0)	2 (4.34)	0.54
Febrile seizure	5 (25)	0 (0)	**0.003**
Drugs	0 (0)	0 (0)	0.9
Family history	3 (15)	11 (23.91)	0.35
Others	4 (20)	13 (28.26)	0.38
**Time of evolution (years): median (interquartile range 25–75)**	28 (14–40)	16 (8–27.5)	**0.028**
**Epilepsy type: n (%)**	**23**	**46**	
Generalized	9 (39.16)	26 (56.52)	0.2
Focal	14 (60.86)	20 (43.47)	0.2
Unknown	0 (0)	0 (0)	0.9
**Location of epileptogenic zone: n (%)**	**23**	**46**	
Frontal	2 (8.69)	4 (8.69)	0.9
Temporal	9 (39.13)	9 (19.56)	0.09
Parietal	0 (0)	1 (2.17)	0.9
Occipital	0 (0)	2 (4.34)	0.54
Insular	1 (4.34)	1 (2.17)	0.9
Mesial	6 (26.08)	0 (0)	**0.0008**
Undetermined	11 (47.28)	29 (63.04)	0.3
**Seizure type: n (%)**	**23**	**46**	
Generalized	9 (39.13)	28 (50)	0.12
Focal impaired awareness	12 (52.17)	15 (32.60)	0.12
Focal aware	2 (8.69)	3 (6.52)	0.9
**Neuroimaging: n (%)**			
Magnetic resonance imaging (n = 61)	**22**	**39**	
Abnormal	12 (54.54)	14 (35.98)	0.18
Normal	10 (45.45)	25 (64.10)	
Computed tomography (n = 22)	**5**	**17**	
Abnormal	0 (0)	6 (35.29)	0.26
Normal	5 (100)	11 (64.70)	
Electroencephalogram (n = 62)	**22**	**40**	
Abnormal	18 (81.81)	26 (65)	0.24
Normal	4 (18.18)	14 (35)	
**Etiology n (%)**	**23**	**46**	
Symptomatic	10 (43.47)	17 (36.95)	0.61
Idiopathic/Undetermined	13 (56.52)	29 (63.04)	

Note: Other risk factors include central nervous system infection, pregnancy complication, birth complication. The *p* -values in bold indicate statistical significance ( *p* < 0.05).

### General population

A comparison of personal history between the two groups showed that history of febrile seizures was more frequent in patients with DRE ( *p* = 0.0018). Regarding the type of seizure and type of epilepsy, focal epilepsy was more frequent in patients with DRE (63.63 vs. 43.58%, *p* = 0.039). Focal seizures with altered consciousness were also more frequent in patients with DRE (54.54 vs. 34.61%, *p* = 0.0371). When comparing the usefulness of neuroimaging studies, in patients with DRE, abnormal magnetic resonance imaging (MRI) was more frequent than in those who responded to medication (61.90 vs. 35.38% respectively; *p* = 0.0098).

Additionally, the usefulness of MRI was evaluated considering abnormal findings, dividing epilepsy into focal or generalized in both drug-resistant epilepsy patients and nondrug-resistant epilepsy. Regarding focal epilepsy, we found that abnormalities were more frequent in patients with drug-resistant focal than with nondrug-resistant disease (18 vs. 16 patients; *p* = 0.43). On the other hand, when comparing pathological findings in patients with generalized epilepsy, we found that these were more frequent in patients with the drug-resistant than with nondrug-resistant condition (57.14 vs. 20%; *p* = 0.01).

Regarding brain computed tomography (CT), the statistical difference was not significant. The finding of epileptiform activity on electroencephalogram (EEG) also did not show significant differences. The findings on neuroimaging studies revealed that symptomatic etiology was the main cause in patients with DRE (56.81%); this result was statistically significant ( *p* = 0.036).

Mesial temporal lobe epilepsy occurred only in patients with DRE. Moreover, patients with DRE had a longer period of evolution, meaning that their diagnosis was earlier than in patients responding to ASD (22 vs. 16 years, *p* = 0.0041).

### Characteristics grouped by sex

In total, 53 patients were male. The presence of personal history was more frequent in patients with DRE (83.36 vs. 53.57%, respectively; *p* = 0.016). There was no evidence that any particular personal medical history was associated with a higher incidence of drug resistance. As in the general population, epilepsy and focal seizures with loss of consciousness were the main types. There was no difference in the location of the epileptogenic focus. When assessing the etiology in the male population, symptomatic etiology predominated over idiopathic etiology (71.42 vs. 34.37%, *p* = 0.011). As a result, MRI abnormalities were more frequently found. Also, the presence of epileptiform discharges on EEG was more frequent in patients with DRE (13 vs. 21 patients respectively, *p* = 0.9). Finally, the time of evolution was longer in patients with DRE, which would imply that their diagnosis was at an earlier age (22.33 vs. 14.83 years, *p* = 0.04), as shown in Table 2 .

In females, febrile seizure was only present in patients with DRE (5 patients with DRE vs. 0 patients with n-DRE, *p* = 0.003). Focal epilepsy predominated in the DRE population, while generalized epilepsy predominated in the female with n-DRE. When considering the location of the epileptogenic focus, mesial sclerosis was present only in the drug-resistant population, being a significant finding. Furthermore, in patients with DRE, the time of evolution was longer (28 vs 16 years respectively; *p* = 0.028). ( Table 3 ).

After performing a univariate study, the variables associated with increased risk of DRE were: febrile seizure (OR: 9.64; 95%CI: 1.97–46.99), symptomatic etiology (OR: 2.34; 95%CI: 1.10–4.99), focal epilepsy (OR: 2. 26; 95%CI: 1.05–4.82), focal seizures with loss of consciousness (OR: 2.26; 95%CI: 1.06–4.82), abnormal MRI (OR: 2.96; 95%CI: 1.32–6.63), and years of evolution (OR: 1.05; 95%CI: 1.01–1.09).

### Multivariate analysis according to sex

When evaluating the characteristics of DRE by means of a multivariate analysis according to sex, it became evident that the male sex showed four factors that were related to a higher risk: personal history (OR: 7.82; 95%CI: 1.54–39.51), time of evolution (OR: 1.05; 95%CI: 1.01–1.106), MRI with abnormal findings (OR: 4.40; 95%CI: 1.26–15.41), and symptomatic etiology (OR: 4.77; 95%CI: 1.44–15.77). For females, only time of evolution was a risk factor for DRE (OR: 1.04; 95%CI: 1.00–1.08), as shown in Table 4 .

**Table 4:** Multivariate analysis of risk factor adjusted by sex

Variable	Odds ratio	95%CI
**Male sex**		
Time of evolution (years)	1.05	1.00–1.106
Personal history	7.82	1.54–39.51
Abnormal magnetic resonance imaging scan	4.40	1.26–15.41
Symptomatic etiology	4.77	1.44–15.77
**Female sex**		
Time of evolution (years)	1.04	1.00–1.08

Notes: Personal history: febrile seizure, traumatic brain injury, central nervous system infection, pregnancy complication, birth complication, and family history.

### Role of years of evolution

**Figure 1:**
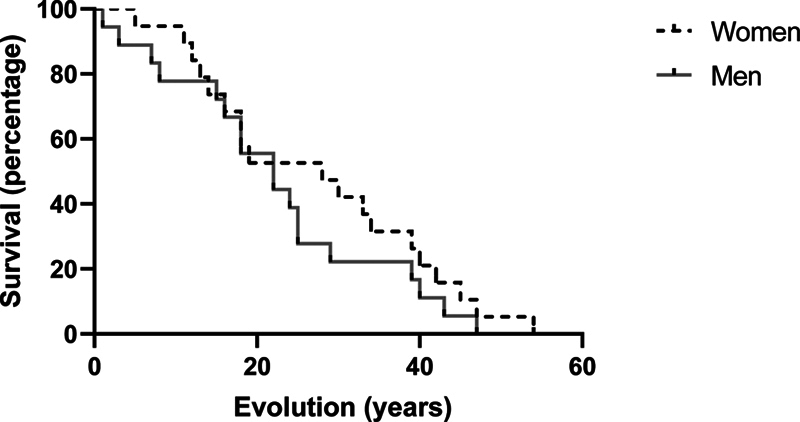
Kaplan-Meier curve. Progression of drug-resistant epilepsy according to years. Median time of progression for men was 22 years, and 28 years for women (log-rank test; *p* = 0.9).

The Kaplan–Meier curve ( Figure 1 ) was used to assess the role of cumulative years of disease evolution in the development of drug-resistant epilepsy. The median time to DRE was 22 years for men and 28 for women; however, this difference was not statistically significant (log-rank test, Mantel–Cox, *p* = 0.90).

## DISCUSSION

Drug-resistant epilepsy is a disease with a reported incidence of approximately 30%, varying according to the definition used. According to the 2010 ILAE definition, 4 the incidence in Argentina was shown to be slightly higher than the described (36%). In agreement with Savic et al., 16 epilepsy was slightly more frequent in women (69 women vs. 53 men), which contradicts the meta-analysis by Kotsopoulos et al. 2 that reports a slight difference in incidence, being higher in men due to greater exposure to risk factors, such as traumatic brain injury, stroke, or CNS infections.

At the population level, recent analyses from the global burden of disease (GBD) study have highlighted a substantial and persistent burden of epilepsy in Latin America and the Caribbean, with relevant differences according to sex. 17 The 1990 to 2019 GBD analysis showed that, although the overall prevalence and disability-adjusted life years (DALYs) attributable to epilepsy have declined over time, epilepsy continues to represent a significant public health challenge in the region, particularly in low- and middle-income countries. It is important to note that specific patterns were observed according to sex, reflecting differences in exposure to risk factors (such as alcohol consumption, access to medical care, and disease outcomes). However, these large-scale epidemiological studies do not address clinical determinants of drug-resistance at the individual level. In this context, our findings contribute complementary evidence by identifying sex-specific clinical and etiological factors associated with drug-resistant epilepsy in a hospital-based cohort from a middle-income country.

In agreement with the characteristics of a previous cohort reported by our team 18 and other authors, 19 20 21 22 we found that a history of febrile seizures is a risk factor, which could be due to its relationship with mesial sclerosis, which is a frequent etiology of DRE. Also, epilepsy and focal seizures with loss of consciousness were associated with an increased risk of DRE in agreement with those reported by Li et al. 22 and Tripathi et al. 23 However, studies by Karaoğlu et al 2.1 and Yu et al. 24 established that the risk is higher when patients suffer multiple seizure types and not only focal seizures.

In our series of patients, both secondary cause and abnormal MRI were significantly increased in patients with DRE. This finding is consistent with that reported in multiple studies, 20 23 25 except for Wang et al. 26 who reported that, while symptomatic etiology was a risk factor, neuroimaging was not. This was because, in his meta-analysis, most of the articles analyzed were from pediatric and adolescent populations. In contrast to other studies, 22 25 26 27 we did not find evidence that the finding of epileptiform activity on EEG, whether focal or generalized, increases the risk of DRE. Finally, it should be considered that the longer the time since diagnosis, the greater the risk of DRE. This is related to the fact that some etiologies that are diagnosed earlier in life (e.g. mesial sclerosis) are more often drug-resistant. Additionally, the evolution of epilepsy, especially when seizure control is not achieved in the early years, constitutes a RF. 20 21 24 28

Regarding DRE and sex, after performing a multivariate analysis, we found that RFs differ according to the sex of the patient, with sex itself not being an RF. In the case of male population, the main cause was symptomatic, being a risk factor for the development of drug resistance. In contrast, in females, idiopathic causes predominated. Our findings are consistent with multiple studies in the literature. 19 20 21 22 29 For example, the study conducted by Li et al., 30 which described that male sex was a risk factor (OR: 2.66) mainly due to higher epilepsy severity and temporal lobe involvement. However, Cepeda et al. 31 reported that women had a higher relative risk (RR) than men (RR: 1.27) possibly related to the hormonal influence on the metabolism of ASDs. Even so, a meta-analysis conducted by Fayad et al. 27 showed that in patients with idiopathic epilepsy or juvenile myoclonic epilepsy (JME), 36.6% of them were drug-resistant (incidence varied from 2.4 to 62%), with the risk of DRE being related to a history of febrile seizures, pathological findings on EEG, the evolution of epilepsy with childhood absences, and psychiatric disorders. Nevertheless, this study did not demonstrate that sex is a risk factor, even though JME is more common in women (ratio 3:1).

When considering neuroimaging studies, pathological findings were a risk factor for males. This is related to a higher incidence of them, because secondary causes (stroke, post-traumatic epilepsy) predominate in the male population.

Finally, although the time of evolution of drug-resistant epilepsy was different according to sex, it was longer in patients with drug-resistant epilepsy in both sexes, being the only medical history that constituted a risk factor in the female population.

Our study has limitations. First, the population in our study is small compared to other studies. Also, since it was done on adult patients, not all of them had been attended to previously in the pediatric service at the time of diagnosis, which means we may not know their full medical history. The limited sample size, particularly in sex-stratified subgroups, reduces statistical power and increases the risk of both type I and type II errors.

In conclusion, although the overall frequency of drug-resistant epilepsy was similar between sexes, relevant sex-specific differences in risk factors were identified. Considering sex at the time of epilepsy diagnosis may improve early risk stratification and help identify patients, particularly men, who could benefit from closer monitoring and earlier therapeutic interventions. A sex-specific approach may therefore support more individualized and equitable epilepsy management.
